# Experiences and effects of psychiatric stigma: Monologues of the stigmatizers and the stigmatized in an African setting

**DOI:** 10.3402/qhw.v10.27954

**Published:** 2015-06-17

**Authors:** Catherine O. Egbe

**Affiliations:** Psychology, School of Applied Human Sciences, University of KwaZulu-Natal, Durban, South Africa

**Keywords:** Stigma and discrimination, mental illness, service users, service providers, stigmatizer, stigmatized, health and well-being

## Abstract

People with mental illness (PWMI) are faced with a number of social and health-related challenges especially stigma and discrimination which tend to have negative effects on their lives. This paper presents narrative monologues portraying the experiences and effects of psychiatric stigma and discrimination on PWMI in South Africa. These narratives voice out the concerns of the stigmatizers (specifically family members and significant others of PWMI) and the stigmatized in a poetic fashion. The society is still not very sympathetic to the plights of PWMI and this affects their general health and well-being. Traditional beliefs and prejudice still drive public attitude towards PWMI especially in African settings. These narratives presented in a poetic fashion in this paper highlight some salient issues relating to the experience and effects of stigma and the desires of PWMI to be treated with love and respect and helped to lead healthy normal lives.

Stigma has been described as a social construct, which is used to define a group of people with characteristics that are perceived as discreditable. Goffman (1963) explained that the people stigmatized are usually perceived as possessing these characteristics that make them to be regarded as tainted and less desirable by other members of the society who are not possessing such attributes.

Stigma compounds the problems faced by those with mental disorders as it leads to strained relationships, depression, social exclusion, low self-esteem, unemployment (Corrigan & Shapiro, 2010; Link & Phelan, 2006; Link, Struening, Neese-Todd, Asmussen, & Phelan, 2014; Sartorius, 1998; Scambler, 1998), poorer health outcomes, and reduced well-being (Rüsch et al., 2014). Studies have shown that stigma has led to many people refusing to seek help for themselves or their relatives and friends who are suffering from mental illness (Kim, Thomas, Wilk, Castro, & Hoge, 2010; Parle, 2011). According to Pinfold, Thornicroft, Huxley, and Farmer (2005), p. 123) “psychiatric stigma tend to make it more difficult for people with mental health problems to benefit fully from available and improved treatment programmes therefore hindering recovery and reintegration.” Stigma, therefore, leads to a reduction in the quality of life of the stigmatized (Hatzenbuehler, Phelan, & Link, 2013).

Globally, there has been a call for interventions to reduce psychiatric stigma and discrimination due to the negative effects it has on the health and well-being of people with mental illness (PWMI), their family members, friends, and caregivers (Collins, Wong, Cerully, Schultz, & Eberhart, 2012; Corrigan, 2004; Thornicroft, 2006).

The aim of this study was to explore the experiences and effects of psychiatric stigma among mental healthcare services users and healthcare providers. This paper, however, presents these findings mainly in a poetic fashion. In these monologues, the experiences of the stigmatized are presented based on the narratives of stigma by PWMI and those who have witnessed PWMI being stigmatized or discriminated against.

The narratives presented in this paper in a poetic fashion highlight some salient issues around the reasons for stigma and the desires of PWMI to lead lives free of these negative experiences and effects of stigma and discrimination.

Three related problems associated with stigma include: *the problem of knowledge* which relates to ignorance; *the problem of attitudes* which relates to prejudice, and *the problem of behaviour* which relates to discrimination (Thornicroft, Brohan, Kassam, & Lewis-Holmes, 2008). These three concepts by Thornicroft et al., which explain stigma, form the conceptual framework upon which the experiences of stigma by service users are elucidated in this paper. This conceptual framework adopted for this study also stems from the work of Goffman cited in Castro and Farmer (2005), p. 54), which defined stigma as “the identification that a social group creates of a person (or group of people) based on some physical, behavioural, or social trait perceived as being divergent from group norms.” The society's perception of service users’ behaviour as being divergent from group norms though borne out of ignorance, informs the discriminatory behaviour and attitude directed at people with mental disorders.

## Methods

This study formed part of the formative stage of the Programme for Improving Mental health care (PRIME) research project in South Africa. PRIME is a research consortium, which is aimed at providing evidence on the implementation and scaling up of treatment programmes for priority mental disorders in five low-and middle-income countries across Africa and east Asia, which include Ethiopia, India, Nepal, South Africa, and Uganda (Lund et al., 2012).

The formative research of PRIME-South Africa focused on exploring the experiences of stakeholders involved in providing and receiving mental health care in primary healthcare settings within the study area in South Africa. A paper has already been published which presented the findings highlighting the types and forms of stigma and discrimination, experiences of externalized stigma, causes of psychiatric stigma, impact of stigma on service users, and participants’ suggested interventions to curb stigma (Egbe et al., 2014). This paper, however, attempts to further explicate the experiences of stigma and discrimination with a view of giving a succinct description of how PWMI are affected by these stigmatizing attitudes, behaviour, and perception (Thornicroft et al., 2008) directed at them. The major feature of this paper is the presentation of the poems; “monologues of the stigmatizers” and “the stigmatized to the stigmatizers” written to convey the findings of this study.

### Study design and study site

This study adopted a qualitative research approach. It was conducted in the Dr Kenneth Kaunda District (KKD) located in the North West Province of South Africa. The KKD is predominantly urban but with about 10% of its population being rural. The population of the KKD is about 632,790 (Lund et al., 2012). Two of the four sub-districts in the KKD were involved in the study. These were the Matlosana and Tlokwe sub-districts, both of which are urban locations with primary healthcare facilities, a specialist in-patient mental health facility and regional hospitals.

KKD was chosen as the PRIME-South Africa's study site with the approval of the South African Department of Health because it is a pilot site for the South African re-engineered primary healthcare system (Matsoso & Fryatt, 2013).

### Sample and sampling technique

A total of 77 participants were purposively sampled from primary healthcare settings and Non-Governmental Organizations (NGOs) facilities serving mental healthcare service users in the study area. The participants include: 32 healthcare service providers and 45 mental health service users with depression, maternal depression, schizophrenia, and bipolar disorder. The sample thus included various categories of mental healthcare service users and healthcare service providers. Service providers include 10 professional nurses, 20 lay counsellors, and 2 auxiliary social workers; service users consist of 20 women with maternal depression, 15 HIV-positive patients with comorbid depression, and 10 persons with severe mental disorder (schizophrenia/bipolar disorder).

Convenience sampling (with the aid of clinic registers and recommendation from the North West Mental health society) was used in the recruitment of participants with severe mental disorders, while other service users were recruited from the waiting rooms of three primary healthcare centres within the study area. Convenience sampling was used to find participants who meet the exclusion and inclusion criteria for the PRIME-South African study and due to the integration of all categories of service users who seek care at primary health centres in South Africa. Mental health service users were expected to have confirmed diagnosis of any PRIME's priority mental health conditions, that is, depression, maternal depression, and schizophrenia/bipolar and should be mentally stable enough to participate in the study. Participants with schizophrenia/bipolar were judged suitable to participate by auxiliary social workers/social workers from the South African mental health society. All participants were above the age of 18 years. Table I presents more details on the study participants and procedure.

**Table I:** Summary of study participants and procedure.

	(Depression comorbid with HIV)	(Schizophrenia-service users and caregivers; Aux. social workers)	Maternal depression	Nurses, lay counsellors
Sample size	15 service users with depression co-morbid with HIV +	10 service users and 10 caregivers +2 auxiliary social workers	20 women diagnosed with maternal depression	10 nurses; 20 lay counsellors
Where were they sampled from?	PHC facility in DRKKD	Clinic and Mental Health Society Klerksdorp	PHC facility in DRKKD	Their respective clinics; Grace Mokhomo, Majara Sephapo, Kanana, Orkney
Sample description (include eligibility criteria)	HIV+ patients who met the diagnostic criteria for major depressive disorder. Participants over age 18 participants, were not pregnant at the time, and had not delivered a baby in the past 5 months. Were diagnosed as HIV+ and did not require urgent medical attention	Service users—schizophrenia/bipolar diagnosis, over 18, able to participate in interview Caregivers—over 18, primary caregiver for person with schizophrenia/bipolar disorder	20 women over the age of 18 attending postnatal clinics Services who met the diagnostic criteria for major depressive disorder and whose infant was between 6 weeks and 12 months old	Purposive volunteer sampling was used Nurses: professional nurses were requested to do interviews and those available and willing were interviewed Lay counsellors: all lay counsellors in all four clinics were requested to do the focus group interviews. The interviews were done for those who were present on the day of the interview
Average time period per interview	50 min	45 min–1 h	50 min	±45 min
Where was the interview conducted?	At the facility	Mental Health Society Offices or Clinic	20 at the facility. 10 follow-up interviews in the home	Nurses and counsellors—onsite, Facility managers—at an eatery
Informed consent	The study was explained to the patients and informed consent was obtained	Informed consent forms signed by participants after the study was explained to them	The study was explained to the patient and informed consent was obtained	Consent forms signed by all participants
Were participants compensated for their time? How much or what was given to them?	Yes. R50-00 vouchers from a local supermarket	Yes. R35 vouchers from a local supermarket	Yes. R50-00 vouchers from a local supermarket	Participants were not compensated for their time. The facility managers were interviewed over lunch
Who conducted the interviews?	A clinical psychologist	2 clinical psychologists	2 clinical psychologists	1 clinical psychologist and 1 research psychologist
Language the interview was conducted	Setswana	18 Setswana; 1 English	Setswana	Setswana
Interpreter used? (Yes/no)	No	No	No	No
Procedure for first round analysis	Guided thematic content analysis was used. Transcripts were analysed using the NVivo software	Thematic content analysis was used aided by the NVivo software	Thematic content analysis was used. Transcripts were analysed using the NVivo software	Thematic content analysis was used aided by the NVivo software

Source: Egbe et al. (2014).

### Data collection

A total of four focus group discussions (with two to seven participants per group) involving 19 lay counsellors and 58 individual interviews were conducted. The focus group discussions (Berg, Lune, & Lune, 2004) were guided by the same interview guide used for the individual interviews. Participants chose to be interviewed in their preferred language. All interviews with the exception of one were conducted in Setswana, which is the predominant language spoken in the study area. Only one interview was conducted in English language. Interviews were voice recorded and were then transcribed and translated. All interviews lasted between 45 min and 1 h, each guided by semi-structured interview schedules for each category of participants, which were available in two versions; Sestwana and English language. The interviewer explained the study to each participant in their preferred language after which they signed the informed consent form as an indication of their voluntary participation in the study. Participants’ response to the following questions formed the data used for this paper; service providers: (1) What is your understanding of mental disorders (depression, maternal depression, psychotic disorders)? (2) Have you been a witness to incidents where a mental health patient was being disrespected, ignored, or discriminated against because of their condition? If yes, can you describe this experience—what happened? Service users: Have you been ill-treated before (at home, in your community or in the clinic)? If yes, how does this ill-treatment make you feel?

### Data analysis

All audio recordings of the interviews were transcribed. The one interview conducted in Setswana was then translated to English language and back translated to ensure validity of the data. Back translation ensures the semantic equivalence of data of this sort (Chen & Boore, 2010). Thematic analysis with the aid of the software NVivo 10.1 was carried out on the data following the steps outlined by Braun and Clarke (2006), which include: (1) familiarization with the data; (2) generating initial codes; (3) searching for themes; (4) reviewing themes; (5) defining and naming themes, and (6) producing the report. The NVivo software was used in generating codes from the data (some of which were preidentified using the interview guide) and organizing these codes into various themes.

Two rounds of analysis were carried out in this study. The first round was carried out by four PRIME-South Africa's researchers (including the author) using thematic analysis. Themes which emerged from this round included stigma and discrimination and other broader issues being investigated as part of the PRIME-South Africa's project. The data on stigma and discrimination were again subjected to thematic analysis by the author of this paper, bearing in mind specific aspects of stigma and discrimination including, types, causes, experiences, effects, and suggested solutions to curb the stigmatization and discrimination against PWMI.

The results represented in this paper form part of the results from the second round of data analysis with themes focusing on the experiences and effects of stigmatizing and discriminatory attitudes by PWMIs as narrated by PWMIs themselves and healthcare service providers who witnessed such attitudes in the health facilities, their homes, or communities. In composing narrative poems to explicate the results in this study, the author sometimes inferred meanings based on the narrative accounts of the participants.

### Ethical consideration

The North West Provincial department of health and the University of KwaZulu-Natal Research and Ethics Board gave permission for the conduct of this study with ethical clearance number HSS/0880/011. Participants were given an informed consent form which carried information about the study, rights of participants, and assurance of confidentiality and anonymity of participants’ responses and identity, respectively. The contact details of the principal investigators of the study were also provided in the informed consent form. In addition, interviewers verbally explained the content of the informed consent form to participants before it was signed by both participants and their interviewers as proof of a voluntary participation in the study. Participants’ identities were protected by the use of code names.

## Results

As mentioned earlier, the results presented in the form of these monologues are based on the themes: experiences of stigma and effects of stigma on service users.

The poems presented in this section attempt to give voices to some of the narratives in the qualitative data analysed. They express also the confusion faced by friends and family members in their bid to make sense of the perceived deviation of their loved ones from their society's norms (Castro & Farmer, 2005) as they notice the change in their attitudes and actions. These friends and family members, therefore, seek help from any possible source to enable their loved ones to be restored to the society. One source of treatment for PWMI in the setting where this study was carried out is the “*sangoma*.” In South Africa, a “*sangoma*” is an African traditional healer who is believed to possess the powers to diagnose and heal the sick (Cumes, 2013). These *sangomas* are often the first port of call among the black Africans for the healing of persons with mental illness.

In the poems presented in this paper, masculine and feminine characters have been portrayed and used intermittently, not because they represent the gender of specific participants but because the author wishes to draw the reader's attention to the fact that mental illness and psychiatric stigma have no gendered boundary. Some verbatim narratives of service users and service providers’, which informed the composition of these poems, are presented.

The “monologues of the stigmatizers” captures the attitude of people who stigmatize and/or discriminate against PWMI as narrated by service users themselves. Healthcare professionals also narrated stigmatizing actions they had witnessed as presented in these narratives.

They would tie her leg to the tree and she won't be able to walk, and most of the time they wouldn't bath her she was dirty … when she tries to talk they would ask why she is talking because she is crazy. (Lay counsellor-LCO1)

Many people are afraid to come to the clinic because they get shouted at there. (Service User-DH13)

You find that in the community that this man has a mental health problem and you find that children in the street would run after him calling him names. (Lay counsellor-LCMS6)

Service users also described how they were considered to be faking the state of their health as expressed in the narrative below.

My family does not understand because they say I'm faking it and they also say I sleep too much. (Service User-S2)

Various other explanatory models were mentioned by participants, which also influenced where the community and family members sought for help for their relatives who suffer from mental illness. The last stanza of the “monologues of the stigmatizers” presents the various treatment options used by people in this setting. In trying to make a decision about which model of care will bring about the much desired healing of their relative or ward, caregivers and family members weigh their options with the possible causes in mind. This implies that they would seek help from the option that best suites what they think is responsible for the problem. A participant's narrative is presented describing the option of seeking help from the “*sangoma*” due to the traditional explanatory model that mental illness is caused by witchcraft.

Other families do that because they believe the person would have been bewitched. They take them there (to the sangomas) because they believe there are some people behind the illness. (Lay counsellor-LCK1)

## Monologue of the stigmatizers

Let's tie him to a tree

Let's keep her away from our friends

They will mock us when she acts weird again

How else can we avert this shame?

He pretends to be sick …

… but nothing seems to be wrong with her physically!

Is it because he just wants to be weird?

What kind of an illness can she possibly have?

‘cos this surely is very abnormal!

Yet he looks very normal!

Oh she is just pretending!

May be she wants attention …

… I'm not gonna give her that

He may really be sick you know …

I'm not sure what to believe anymore

What they say or what I think?

But she may just have been bewitched …

Or our ancestors need him to serve them

Or a demon may have taken charge of her

But who can help him now?

Where can we get help for her?

A church? Yes … if she has a demon, but I don't know

A *sangoma?* Yes if our ancestors want him, but I really don't know

A witchdoctor? Yes if she is bewitched, but how do I know?

A hospital? Not sure the doctors really would know

What then can we do to solve this problem?

We really want her to come back to us

We don't want to lose him

Or be ridiculed or isolated because of her

How can we come out of this?

The poem titled “the stigmatized to the stigmatizers” is also based on the experiences of stigma by PWMI or the narrative accounts of healthcare providers who have witnessed PWMI being ill-treated. It is, however, expressed in the voice of the stigmatized and also portrays the effects the stigmatizing and discriminatory attitude, perception, and behaviour directed at PWMI by their family members, friends, and community members because of their mental health status. Some of the narrative accounts, which informed these poems are presented here. Service users mentioned being mocked and ill-treated by friends and neighbours.

It was about 4 months ago when I was with a friend, we were at his place, I was watching TV and he told people that I'm a lunatic. Even my uncle who is younger than me was with girls one time and he made fun of me and told them I'm a lunatic. They discriminate against me by teasing me and saying all those things. (Service User-S9)

Do you think they understand your illness? (Interviewer)

No they don't because one of them once asked me that “what is happening to you because to me you are just fine.” (Service User-S4)

They ill-treat me to make themselves happy and show other people that they can mock me. They just want me to be the centre of attention so that people can assess me and check my weak and strong points. (Service User-S5)

## The stigmatized to stigmatizers

Oh no! Why do you treat me so?

Children throwing stones at me

My family no longer loves me so

I'm tied up like an animal gone wild

Oh why do you beat me up so?

What have I done to deserve this?

People point fingers at me as I walk the streets

I hear them whisper as I walk the streets

Some even let me hear it

Blaming me for being “weird,” as they call it

But I understand not what they say sometimes

Though their expressions capture it all sometimes

Am I still useful to this society?

Am I still useful to my family?

I know not anymore …

Their faces say a thousand words

And they say I have changed …

… but who really has changed?

They treat me right no more

And I really know not why

They say I'm not fit to work

They say I'm not fit to get married

They say I don't make sense anymore

They say I keep silent for too long

They seem to say I'm not good enough

At least … not anymore

I wonder what I can do to change the way they see me

‘cos for now, they seem not to love me anymore

## Discussion

The beauty of using qualitative research methods to explore a phenomenon can be seen in the richness of the qualitative data and the diversity of its sources. While a quantitative method would have yielded its own unique data, qualitative research allows for the researcher to interact with the participant and the data so much that the words spoken by the participants can be presented in the best way that could capture the attention of those who may help to redress the situation as can be found in the case of psychiatric stigma. This is the motivation for the poetic narration of the experiences of the stigmatizers and the stigmatized as well as the effects of psychiatric stigma on the stigmatized in this paper.

The findings described in the “monologues of the stigmatizer” confirm the assertion by Carr, Halpin, and Group (2002) that people with psychotic illnesses are usually victims of violence. This also confirms the definition of stigma as “a discrediting social difference that yields a spoiled social identity” resulting in widespread societal disapproval (Bos, Pryor, Reeder, & Stutterheim, 2013, p. 1). PWMI are treated like citizens who lack some form of characteristics, which should qualify them to be treated like any other member of the society. Many stigmatizers attribute the stigmatizing conditions in these persons to a fault of the stigmatized. Thus, PWMI are sometimes blamed for the state of their mental health as found in this study.

The poem “the stigmatized to the stigmatizers” relay service users experiences of stigma and discrimination from family, friends, and members of the public. This finding is in line with the findings revealing that PWMI are often not regarded as desirable members of the community and worse still as “not quite human” (Goffman, 1963). In the views of Bos et al. (2013, p. 1), stigma manifests as “… aversion to interaction, avoidance, social rejection, discounting, discrediting, dehumanization, and depersonalization of others into stereotypic caricatures.”

Psychiatric stigma though a long existing type of stigma, still has debilitating effects on the people living with mental illness and on their health and well-being (Hatzenbuehler et al., 2013; Rüsch et al., 2014). Stigma reduces help seeking and worsens the state of health of the victims (Clement et al., 2015; Corrigan, 2004). It has been well documented that many actions of people who perpetuate stigma in the society are borne out of ignorance and prejudice (Thornicroft, 2006). It is the author's desire also that the voices of the stigmatizers would be heard by key stakeholders as well, so that they would provide the much needed educational and psychosocial interventions and problem-solving skills for these categories of persons who are largely burdened with a poor understanding of the manifestations of mental illness (Dixon et al., 2014).

For the sake of improving the health and well-being of PWMI as well as that of their caregivers, family members, and the society at large, there is the need for all hands to be on deck to design and implement programmes and policies that will reduce psychiatric stigma at all levels in the society (Rüsch et al., 2014).

## Conclusion

Qualitative methods have been described as an invaluable means of exploring the experiences of healthcare service users (Smith & Firth, 2011). Working with the qualitative data collected from mental health service users and their healthcare service providers formed the bases of this paper which presented in a poetic fashion, the words and some inferred thoughts of the stigmatized and the stigmatizers. It is hoped that the voices of the stigmatized would be heard by policy makers and programme implementers to guide them in providing quality services and anti-stigma interventions that can uplift the lives of those suffering from mental illness.
